# Monotherapy or combinations? Intravenous vitamin C in sepsis and septic shock: An umbrella review of 31 systematic reviews

**DOI:** 10.1371/journal.pone.0351072

**Published:** 2026-07-01

**Authors:** Víctor Juan Vera-Ponce, Jhosmer Ballena-Caicedo, Lupita Ana Maria Valladolid-Sandoval, Fiorella E. Zuzunaga-Montoya, Renzo Acosta-Porzoliz, Oriana Rivera-Lozada, Mario J. Valladares-Garrido

**Affiliations:** 1 Facultad de Medicina (FAMED), Universidad Nacional Toribio Rodríguez de Mendoza de Amazonas (UNTRM), Amazonas, Perú; 2 Facultad de Medicina Humana, Universidad Peruana Cayetano Heredia (UPCH), Lima, Perú; 3 EpiHealth Research Center for Epidemiology and Public Health, Lima, Perú; 4 Vicerrectorado de Investigación, Universidad Señor de Sipán, Chiclayo, Perú; 5 Oficina de Inteligencia Sanitaria, Red Prestacional EsSalud Lambayeque, Chiclayo, Perú; University of Ghana College of Health Sciences, GHANA

## Abstract

Intravenous (IV) vitamin C has been proposed as an adjuvant therapy in sepsis/septic shock due to its biological plausibility and safety profile, but the proliferation of reviews has not resolved its clinical utility. To synthesize the evidence on IV vitamin C (monotherapy, HAT—hydrocortisone+vitamin C+thiamine—and vitamin C+thiamine) in adults with sepsis/septic shock, prioritizing 28–30-day mortality. Umbrella review of systematic reviews and meta-analyses (MEDLINE/PubMed, Embase, Scopus, and Web of Science, without language restriction). Quality was assessed with AMSTAR 2, overlap with CCA, and when available, TSA and component/network meta-analysis (CINeMA). Certainty of evidence was graded using GRADE with an anchor estimator per outcome and regimen. Thirty-one reviews were included (30 quantitative: 28 SR/MA and 2 component/network MA; 1 qualitative). Combinations (HAT and vitamin C+thiamine) did not reduce mortality; hemodynamic improvements (small decreases in ΔSOFA and vasopressor hours) were modest, did not translate into survival benefits, and were primarily attributable to the corticosteroid. Monotherapy showed a possible mortality benefit signal under specific conditions (initiation ≤24 h, intermediate dose 25–100 mg/kg/day, 3–4-day courses; more pronounced in sepsis than shock), but with low-to-moderate certainty due to heterogeneity, imprecision, publication bias, and very high overlap among reviews. Combination regimens (HAT and vitamin C plus thiamine) did not reduce mortality; hemodynamic improvements were modest, did not translate into survival benefits, and were primarily attributable to the corticosteroid component. For monotherapy, a possible mortality benefit signal was identified under specific conditions (initiation within 24 h, intermediate dose 25–100 mg/kg/day, 3–4-day courses, more pronounced in sepsis than shock), but overall certainty remains low-to-moderate due to heterogeneity, imprecision, publication bias, and very high overlap among reviews. These findings do not support routine use of IV vitamin C in any regimen; for monotherapy, the identified signal warrants rigorous multicenter trials in well-defined clinical scenarios before any recommendation can be made.

## Introduction

Sepsis and septic shock remain conditions with a high global burden, with an estimated 49 million cases and 11 million deaths annually in 2017, and in-hospital mortality that, in ICU settings, may approach 25–40% depending on severity and comorbidities [1,2]. Following the Sepsis-3 update [3], management remains centered on hemodynamic resuscitation, early antibiotics, and source control; however, the search for adjuvants capable of improving survival remains ongoing. In this context, intravenous (IV) vitamin C emerged as a potential intervention due to its role in catecholamine synthesis, nitric oxide modulation, and its antioxidant effects at the endothelial and immune levels [4].

Early trials and clinical series suggested physiological benefits and, in some cases, survival benefits, particularly when IV vitamin C was administered as monotherapy, at intermediate doses, and in early phases of sepsis [5–8]. Subsequently, combinations such as HAT (hydrocortisone + ascorbic acid + thiamine) and vitamin C + thiamine without steroids were explored [9–13]. However, the accumulated evidence has proven heterogeneous and often contradictory: while several reviews and meta-analyses of pure RCTs support a signal of reduced 28–30-day mortality with vitamin C monotherapy [6,7,9], others with mixed samples (RCTs + observational studies) do not confirm this effect [14,15]. In parallel, three large RCTs have substantially shaped the current debate. LOVIT (n = 872) tested high-dose vitamin C monotherapy (200 mg/kg/day for 4 days) and found no mortality benefit and a possible increase in 28-day mortality and organ dysfunction in the intervention arm [16]. VICTAS (n = 501) evaluated HAT and found no improvement in vasopressor- and ventilator-free days [17]. VITAMINS (n = 216) compared HAT against hydrocortisone alone and found no difference in vasopressor-free days at 7 days, suggesting that the corticosteroid, rather than the vitamin combination, drives any hemodynamic benefit [18]. These results do not necessarily contradict the biological rationale, but they do constrain it: the pharmacological window in which vitamin C may be effective — if it exists — appears narrow and highly dependent on dose, timing, and patient selection.

For combination regimens, the most robust literature suggests that HAT does not reduce mortality and only modestly improves supportive outcomes; for example, Sequential Organ Failure Assessment delta (ΔSOFA) at 72–96 hours and duration of vasopressors, with small magnitude and questionable clinical relevance; furthermore, network component analyses indicate that the glucocorticoid would explain most of the hemodynamic benefit, not vitamin C or thiamine [11,12,19,20]. This dissociation between benefit in surrogate outcomes and absence of effect on survival has motivated the need for a methodologically critical synthesis that distinguishes IV vitamin C monotherapy from combinations, and that separates sepsis from septic shock, as well as dose, timing of initiation, and duration.

Therefore, we conducted an umbrella review (UR) focused exclusively on IV vitamin C-based regimens in adults with sepsis or septic shock, with three main comparators: (1) IV vitamin C monotherapy, (2) HAT, and (3) vitamin C + thiamine. Our objective was to critically describe the existing evidence, assess its methodological quality, and summarize the certainty by outcome, with special emphasis on 28–30-day mortality and supportive outcomes (ΔSOFA 72–96 h, duration of vasopressors).

## Materials and methods

### Study design

UR of systematic reviews, meta-analyses (MA), and (when applicable) network meta-analyses (NMA) on IV vitamin C-based regimens in adults with sepsis or septic shock. This UR was developed following the PRIOR (Preferred Reporting Items for Overviews of Reviews) guidelines for umbrella reviews [21] (see S1 Table), and additionally the methodological framework proposed by Aromataris et al. [22] for this type of study. A completed PRISMA 2020 checklist is provided as Supporting Information to comply with reporting requirements for systematic reviews and evidence syntheses (see S1 Checklist PRISMA)

### Search strategy

Structured searches were conducted in PubMed/MEDLINE, Scopus, Web of Science (including all collections), and EMBASE from the inception of each database until the project cutoff date. Search terms (combined with Boolean operators and MeSH/Emtree equivalents when applicable) included, as a guide: sepsis, septic shock, vitamin C, ascorbic acid, hydrocortisone, thiamine, HAT, randomized, systematic review, meta-analysis, network. No language restrictions were applied (See S2 Table).

### Selection criteria

We included: (1) systematic reviews (SR) with or without meta-analyses (MA) and network meta-analyses (NMA) evaluating intravenous (IV) vitamin C as monotherapy, HAT (combination of hydrocortisone + vitamin C + thiamine), or vitamin C + thiamine in adults (≥18 years) with sepsis or septic shock; (2) studies reporting at least one outcome of interest: 28–30-day mortality (primary), ΔSOFA at 72–96 h (change in Sequential Organ Failure Assessment score), duration of vasopressors, ICU length of stay (ICU-LOS), hospital length of stay (hospital-LOS), acute kidney injury (AKI)/renal replacement therapy (RRT), and adverse events; (3) articles with clear design, explicit inclusion criteria, and reproducible literature search.

We excluded: (1) narrative reviews without reproducible criteria; (2) studies in pediatric or non-adult populations; (3) non-IV vitamin C interventions or combinations not aligned with the focus (e.g., nutraceutical cocktails where the specific effect of vitamin C cannot be separated).

### Selection process and data extraction

Three investigators (VJVP, JJBC, LAMVS) independently performed screening and extraction in duplicate using Rayyan (review screening platform from QCRI). In the first phase (title/abstract), clearly irrelevant records were excluded, and the full-text review applied the inclusion/exclusion criteria. Discrepancies were resolved by consensus or with adjudication by a fourth reviewer (FEZM).

From each review, we extracted: study type (SR/MA/NMA), population, databases and search period, number of randomized controlled trials (RCTs) and non-RCTs, total sample size, intervention (dose, timing, and duration), comparators, reported outcomes with their estimators and precision; registration (e.g., PROSPERO), risk of bias (RoB) tool used for primary studies, publication bias assessment (funnel/Egger), and synthesis methods (random or fixed-effects model; NMA; trial sequential analysis—TSA).

### Table construction

To ensure a homogeneous and auditable recording of the data, a data dictionary and a single template were developed, structured into three main blocks. The first block, “Identification and scope,” was used to record the fundamental information of each study, including a PDF number assigned for initial order and traceability, the reference in Vancouver format, the type of treatment studied (IV vitamin C monotherapy, HAT, or vitamin C + thiamine), as well as the population and clinical setting.

The second block compiled the “Methodological metadata” of each review. This section documented the databases searched, the search period, the designs of the included studies, the number of randomized controlled trials (RCTs) and non-RCTs, the total sample size, the comparators used, and the statistical synthesis model (random/fixed effects or network meta-analysis). Additionally, it was recorded whether a Trial Sequential Analysis (TSA) or a CINeMA (Confidence in Network Meta-Analysis) assessment had been performed, as well as the protocol registration information (e.g., in PROSPERO).

Finally, the third block focused on the “Quantitative results” for key outcomes: 28–30-day mortality, change in SOFA score (ΔSOFA) at 72–96 hours, and duration of vasopressor use. For each of these outcomes, the effect measure was recorded, such as risk ratio (RR), odds ratio (OR), mean difference (MD), or standardized mean difference (SMD), along with its 95% confidence interval (95% CI) and the heterogeneity statistic (I²), also specifying the model used in each review

When explicitly reported by the review, we added operational columns: initiation ≤24 h, duration 3–4 days, dose category 25–100 mg/kg/day vs > 100 mg/kg/day, and sepsis vs shock.

Extraction was performed in duplicate by three investigators (VJVP, JJBC and LAMVS) independently, after a pilot calibration phase (5 reviews), and any discrepancy was resolved by a fourth investigator (FEZM). The process was managed in Rayyan for screening and in a master sheet (auditable format) for data capture; any subsequent corrections left a trace (timestamp, author of change, and reason). When a review presented multiple time horizons (e.g., 28 d, in-hospital, ICU), 28–30 days was prioritized; when multiple models existed (fixed/random), the random-effects declared as primary were extracted. In network/component meta-analyses, we recorded the estimator for the relevant comparison (when available) and network parameters (e.g., consistency/coherence).

### Risk of bias analysis

Methodological quality of each review was assessed with AMSTAR-2 (items 1–16), with special emphasis on critical items: prior protocol (2), list of excluded studies with justification (7), RoB assessment of primary studies (9), meta-analysis methods (11), consideration of RoB in interpretation (13), and publication bias (15) [23]. Three investigators applied AMSTAR-2 independently (VJVP, JJBC and LAMVS) and a fourth resolved disagreements (FEZM). We recorded item-by-item ratings (Yes/Partial/No/Not reported/N/A) and the overall synthesis (High/Moderate/Low/Critically low), which was compiled in S3 Table (with color codes for critical items).

As a complement, we documented how each review assessed RoB of RCTs (e.g., Cochrane RoB 2, Jadad), handling of publication bias (funnel, Egger), treatment of heterogeneity (I², sensitivity analyses), and when present, application of TSA or CINeMA assessment in NMA.

### Certainty of evidence (GRADE)

To rate certainty with GRADE [24], we adopted an “anchor estimator” strategy when multiple reviews reported the same outcome with similar effect measures. Rather than performing a “meta-meta-analysis” or averaging results, we selected a single reference estimator for each critical outcome (28–30-day mortality) and therapeutic regimen (monotherapy, HAT, C + T). This anchor estimator consisted of a single RR/OR/MD/SMD with 95% CI (and I² when applicable) extracted from the most methodologically robust review, prioritizing high AMSTAR-2 quality, strict PICO alignment (28–30-day horizons over alternatives), random-effects model, and TSA availability.

The remaining reviews from the same silo were not statistically combined but served complementary functions to validate consistency of findings, explore subgroup analyses by dose (25–100 mg/kg/day vs > 100 mg/kg/day), timing of initiation (≤24h), duration (3–4 days) and population (sepsis vs septic shock), and perform sensitivity analyses to assess result robustness. GRADE application started from high certainty given these were RCTs synthesized in systematic reviews, with systematic downgrading across five domains: risk of bias (quality of individual RCTs and meta-analysis), inconsistency (I², directionality of effects, and confidence interval overlap), indirectness (inappropriate mixing of populations without stratification), imprecision (wide intervals crossing the no-effect threshold, optimal information size not reached, supported by TSA when available), and publication bias (funnel plot asymmetry or positive Egger test).

Importantly, the GRADE process was executed in duplicate by three investigators (VJVP, JJBC and LAMVS) with a fourth resolving disagreements (FEZM). The final presentation was delivered through SoF tables, separated by regimen (monotherapy, HAT, C + T), and included an anchor estimator (95% CI and I²), a final rating (High/Moderate/Low/Very low), and justification for each adjustment. Critical decisions (e.g., anchor estimator selection when multiple high-quality SRs existed) were recorded in a log with links to the corresponding figure/page in the PDF for peer review traceability.

A sensitivity analysis evaluating the stability of GRADE conclusions when the primary anchor is replaced by the second- and third-ranked reviews by AMSTAR-2 score is provided in Supplementary Material (S4 Table). The direction and certainty category of all conclusions remained unchanged across substitutions.

### Overlap analysis

To avoid double counting and identify redundancy, we quantified RCT overlap between reviews for each “silo” (Vitamin C monotherapy; HAT; Vitamin C+thiamine) using the Corrected Covered Area (CCA) by Pieper et al. [25]. Primary articles were extracted by three investigators (VJVP, JJBC and LAMVS), with discrepancies resolved among themselves.

For each silo, unique RCTs and their appearances in each SR/MA were listed; we applied:


$$\begin{array}{c} CCA=(No-Ns)÷((r\;\times c)\;-\;Ns) \end{array}$$

where No = total number of RCT appearances; Ns = unique RCTs; r = number of reviews; c = unique RCTs. Interpretation was: 0–5% slight, 6–10% moderate, 11–15% high, > 15% very high.

### Statistical analysis

No new meta-analyses or meta-regressions were performed. Quantitative conclusions are based on estimators and TSA reported by included reviews. The objective was descriptive-critical, prioritizing quality (AMSTAR-2), certainty by outcome (GRADE), and consistency among reviews with very high CCA.

## Results

### Study selection

**Fig 1:**
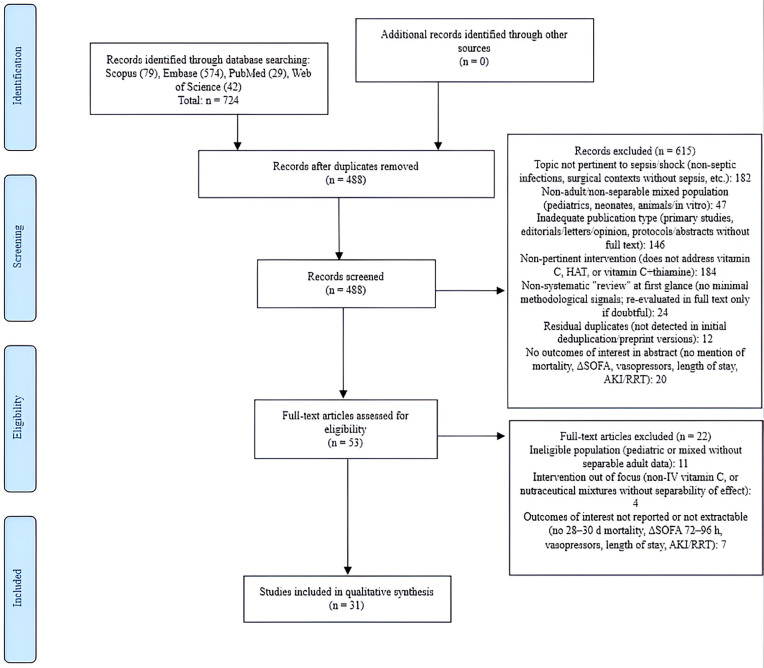
PRISMA 2020 flow diagram of the study selection process.

The search in the four aforementioned databases retrieved 724 records with no additional sources identified through other means. After deduplication, 488 references were retained and screened by title and abstract; of these, 435 were excluded due to clear lack of relevance (e.g., non-adult populations, non-intravenous interventions, primary studies or narratives). Full-text assessment was performed on 53 articles, with 22 excluded for not meeting inclusion criteria (mainly: narrative review without reproducible methods, pediatric population, non-aligned intervention; for example, nutraceutical cocktails without separability of vitamin C effect, or absence of outcomes of interest). Finally, 31 reviews were selected [5–12,14,15,19,20, 26–44]. Fig 1 shows the PRISMA 2020 flow diagram of the study selection process. (Fig 1)

### Main characteristics of studies

Thirty-one reviews on intravenous vitamin C-based regimens in adults with sepsis or septic shock were included: 30 quantitative (28 systematic reviews with traditional meta-analyses and 2 network/component meta-analyses) and 1 qualitative (for methodological support) (See Table 1 and S5 Table). Within the quantitative corpus, we identified three analytical sets (“silos”): IV vitamin C monotherapy, HAT (hydrocortisone + vitamin C + thiamine), and vitamin C + thiamine without steroid. The body of trials feeding each silo is compact: 10 unique RCTs support HAT (VITAMINS, ACTS, VICTAS, ORANGES, Hwang, Chang, Mohamed, Lyu, Hussein, Jamshidi) [10–12,35], 10 unique RCTs support monotherapy (e.g., Zabet 2016; Fowler 2019; Wacker 2022; Rosengrave 2022; Lv 2021; El-Driny 2022) [5–7,37,40], and 7 unique RCTs support vitamin C + thiamine [13,41]. In monotherapy, 28–30-day mortality was the most frequent primary outcome; in HAT and vitamin C + thiamine, ΔSOFA at 72–96 h was often treated as primary and mortality as secondary [10–13]. Seven reviews employed trial sequential analysis (TSA) to anchor interpretation [6,10–13,27,29] and two used component analysis to disaggregate the effect of corticosteroid, vitamin C, and thiamine [20,34]. Comparators were predominantly placebo/standard care; in HAT, several RCTs contrasted against hydrocortisone alone (e.g., VITAMINS), relevant data for interpreting the absence of survival effect [10,34].

**Table 1:** Summary of included systematic reviews grouped by regimen and primary outcome focus.

Regimen	Primary outcome focus	Included reviews (first author, year)	N reviews	Total N (range)	Key result direction	AMSTAR-2 rating
**IV VITAMIN C MONOTHERAPY**	28-30d mortality (primary or sole outcome)	Martimbianco 2022; Scholz 2021; Brown 2022	3	3,133–4,078	Possible benefit in pure RCT subsets (RR 0.60 [0.45–0.80] for Martimbianco); null in mixed RCT+observational designs (OR 0.92 [0.78–1.09] for Brown). Certainty: low-to-moderate.	High (1); Moderate (2)
	28-30d mortality + ΔSOFA / vasopressor hours (co-primary or secondary)	Hung 2023; Lee 2023; Liang H 2023; Liang B 2023; Zeng 2023; Luo 2023; Feng 2021; Li 2021; Zhu 2022; Wei 2020	10	584–3,759	Mortality signal under specific conditions (initiation ≤24 h, dose 25–100 mg/kg/d, 3–4-day courses, sepsis > shock); vasopressor hours reduced in several SRs; ΔSOFA heterogeneous and inconsistent across designs. TSA confirms insufficient sample in most anchors.	High (4); Moderate (6)
**MIXED “VIT C-CONTAINING” REGIMENS**	28-30d mortality (primary)	Chen 2022; Cai 2022	2	1,423–2,985	Null effect across all mortality horizons (ICU, in-hospital, 28d, 90d; OR range 0.84–1.09). Includes RCT + observational designs; interpretation limited by regimen heterogeneity.	Moderate (2)
	28-30d mortality + ΔSOFA / vasopressor hours	Wen 2023; Tariq 2022; Muhammad 2022	3	2,712–3,759	Null overall mortality (OR 0.78–0.87; mixed significance); modest ΔSOFA reduction (SMD 0.26 [0.09–0.42]) and vasopressor shortening reported. Heterogeneous populations and co-interventions limit interpretation.	Moderate (3)
**HAT (HYDROCORTISONE + VITAMIN C + THIAMINE)**	28-30d mortality + ΔSOFA / vasopressor hours	Assouline 2021; Na 2021; Zayed 2022; Wu 2021; Kato 2023; Lu & Mao 2023; Somagutta 2021; Shi & Tie 2020	8	839–1,572 (RCT-based)*	Null mortality across all anchors (RR 0.96–1.05, I² = 0%; TSA confirms futility/insufficiency). Consistent ΔSOFA improvement (WMD −0.69 to −0.92) and vasopressor reduction (WMD −15 to −25 h); effects small and not accompanied by LOS or survival benefit.	High (3); Moderate (4); Low (1)
**VITAMIN C + THIAMINE (WITHOUT CORTICOSTEROID)**	ΔSOFA/vasopressors (primary); 28-30d mortality (secondary)	Ge 2021	1	868	Null in-hospital mortality (OR 1.11 [0.79–1.56], I² = 0%). ΔSOFA improved (WMD 0.83 [0.27–1.38]); vasopressor hours reduced (WMD −17.73 [−29.76; −4.98]). Single SR limits generalizability.	Moderate (1)
**THIAMINE MONOTHERAPY**	28-30d mortality (primary)	Kanchanasurakit 2021; Qian 2020	2	592–645	Null mortality in both SRs (OR 0.96 [0.72–1.28]; OR 0.87 [0.62–1.21]; I² = 0%). No significant ΔSOFA or vasopressor effect. Limited number of RCTs (3–4 per SR).	Moderate (2)
**COMPONENT / NETWORK META-ANALYSIS**	Mortality + vasopressor hours / ICU-LOS	Fujii 2022; Fong 2021	2	9,898–10,257	No mortality benefit attributable to vitamin C or thiamine components (longer-term or short-term). Vasopressor shortening and ICU-LOS reduction consistently explained by the glucocorticoid component (iMD −29.8 h; −1.3 d). CINeMA used for certainty.	High (2)

* Somagutta 2021 includes a large cohort component (N = 67,000); RCT-based N shown for HAT silo. Abbreviations: RCT = randomized controlled trial; SR = systematic review; MA = meta-analysis; NMA = network meta-analysis; ΔSOFA = change in Sequential Organ Failure Assessment score; HAT = hydrocortisone + ascorbic acid + thiamine; TSA = Trial Sequential Analysis; ICU-LOS = intensive care unit length of stay; WMD = weighted mean difference; MD = mean difference; SMD = standardized mean difference; RR = risk ratio; OR = odds ratio; iMD = indirect mean difference; ns = non-significant; NR = not reported. AMSTAR-2 ratings in parentheses indicate number of reviews per category. The complete characteristics table is provided in S5 Table.

### Objectives and doses used

Reviews converged on three clinical questions: whether IV vitamin C monotherapy reduces 28–30-day mortality and improves supportive outcomes; whether combinations (HAT and vitamin C + thiamine without steroid) add benefit over standard care; and which administration conditions (timing of initiation, duration, severity) modulate the effect [5–7,10–13,37]. In parallel, component meta-analyses examined which drug explains hemodynamic changes in combinations, consistently pointing to the glucocorticoid as the main determinant of vasopressor shortening and ICU length reduction, without clear translation into survival when adding vitamin C or thiamine [20,34].

Monotherapy was most frequently administered as 1.5 g every 6 hours (equivalent to 25–100 mg/kg/day) for 3–4 days, with initiation within 24 hours of sepsis recognition; under these conditions, signals of short-term mortality reduction and greater coherence between reviews are concentrated [5,7,36,37]. In contrast, very high doses (≥10 g/day or >100 mg/kg/day) and longer courses showed no reproducible advantage and, in several analyses, attenuated the signal [6,30]. HAT regimens followed relatively uniform protocols (hydrocortisone 50 mg/6 h + vitamin C 1.5 g/6 h + thiamine 100–200 mg every 6–12 h for 3–7 days) and vitamin C + thiamine replicated those doses omitting the steroid; in both cases, favorable effects were limited to ΔSOFA 72–96 h and vasopressor hours of small magnitude, without reproducible impact on survival [10–13].

### Overlap index (CCA)

To quantify trial overlap between reviews in each silo, we calculated the CCA from the lists of RCTs included in each review. In HAT [10–12,28,35], we identified 10 unique RCTs appearing 45 times in total across 5 reviews; with Ns = 10, No = 45, r = 5 and c = 10, the CCA was 0.875 (87.5%), indicating very high overlap. In IV vitamin C monotherapy [5–7,37,40], we counted 10 unique RCTs appearing 40 times in 5 reviews; Ns = 10, No = 40, r = 5, c = 10 → CCA 0.750 (75.0%), also very high. In vitamin C + thiamine, 7 unique RCTs were recorded with 12 appearances in 2 reviews; Ns = 7, No = 12, r = 2, c = 7 → CCA 0.714 (71.4%), equally very high. These values confirm that reviews within each silo essentially synthesize the same body of trials; therefore, in the narrative synthesis we prioritized reviews of higher quality (high AMSTAR-2) and, when applicable, with TSA as main estimators, using the rest for sensitivity analysis and coherence, without additional quantitative aggregation. Inclusion matrices (RCT × review) used for calculation will be attached as supplementary material.

### Methodological quality (AMSTAR-2)

The methodological quality of reviews supporting the main conclusion was predominantly high or moderate (See S3 Table). In monotherapy, several syntheses met critical items (prior protocol, risk of bias assessment, appropriate meta-analytical methods, consideration of bias in interpretation, and formal publication bias assessment) and achieved high AMSTAR-2 [6,7,37]. In HAT, meta-analyses with TSA [10–12] also showed high AMSTAR-2, as did the component/network MA that disaggregated component effects [34]. The remaining monotherapy and combination reviews generally obtained moderate AMSTAR-2 due to a non-critical weakness (e.g., incomplete list of excluded studies or partial reporting of primary study funding) [5,9,13,30,41]. A mini-MA in letter format lacked protocol and presented abbreviated domain assessment, resulting in low rating [43]. Strength and weakness patterns were consistent across silos: when reviews were pure RCTs and registered protocol with justified exclusion list, the rating was high, and when they integrated RCTs + observational studies without clear stratification or without discussing risk of bias impact, the rating descended to moderate [14,15,35].Explicitly, umbrella conclusions were anchored in reviews with high AMSTAR-2 and, when available, with TSA [6,10–12], while those of moderate quality were used for sensitivities (dose, initiation, duration, severity) and external coherence.

### Certainty of evidence (GRADE)

Certainty for IV vitamin C monotherapy on 28–30-day mortality is low-to-moderate: several SR/MA of pure RCTs show coherent relative reductions (RR 0.60–0.75) when the intervention is administered early (≤24 hours), with intermediate dose (around 1.5 g/6 h; 25–100 mg/kg/day) and for 3–4 days [5–7,36]. Certainty is downgraded due to clinical/statistical heterogeneity, publication bias indicators in large meta-analyses (Egger tests), and still moderate sample sizes. Nonetheless, the directional consistency between RCT syntheses supports a greater clinical probability of benefit in the sepsis scenario compared to shock [9]. For surrogates with monotherapy (ΔSOFA, vasopressors), certainty is low, due to variability between studies and elevated I² in several analyses [6,27] (See Table 2).

**Table 2:** Summary of Findings (GRADE): Intravenous vitamin C monotherapy in adults with sepsis/septic shock.

Outcome	Anchor Estimator (95% CI, I²)	Certainty (GRADE)	Notes
28–30-day mortality	RR 0.76 (0.60–0.97); random model (Hung 2023)	Low–Moderate	Coherent with OR 0.51 (0.37–0.69); I² = 0% (Zhu 2022) and RR 0.60 (0.45–0.80); I² = 0% (Martimbianco 2022). Clearer signal with 25–100 mg/kg/day, 3–4 days and initiation ≤24 h.
ΔSOFA 72–96 h	MD −0.05 (−1.69; 1.58) (Zhu 2022) / MD 0.04 (−0.55; 0.63) (Liang 2023) → ns	Low	Clinical/statistical heterogeneity; absence of reproducible pooled effect in pure RCTs.
Duration of vasopressors	MD −27.9 h (−49.8; −5.9); I² = 95% (Zhu 2022)	Low–Moderate	In Lee 2023: MD −0.79 days (−1.24; −0.34) (≈ −19 h); and in Hung 2023: MD −37.75 h (−70.77; −4.73). Effect present but with high heterogeneity between RCTs.
ICU-LOS / Hospital-LOS	SMD −0.33 (−0.87; 0.20) → ns (Feng 2021)	Low–Moderate	No consistent reduction in length of stay observed.
Adverse events	Heterogeneous; no clear increase in pure RCTs	Low	In large meta-analyses with mixed designs, signal of increased AE appears; not in pure RCTs (interpret with caution).

GRADE reasons (monotherapy): • Risk of bias: Small RCTs; variations in blinding. • Inconsistency: Heterogeneity in surrogates, doses, initiation, and duration. • Imprecision: Moderate sizes; wide CIs in some subgroups. • Publication bias: Suggested in large SRs (Egger test). Abbreviations: AE = adverse events; CI = confidence interval; GRADE = Grading of Recommendations Assessment, Development and Evaluation; ICU-LOS = intensive care unit length of stay; MD = mean difference; RCT = randomized controlled trial; RR = risk ratio; SR = systematic review; TSA = Trial Sequential Analysis; WMD = weighted mean difference.

In HAT and vitamin C + thiamine, certainty for mortality is low (stable null effect or insufficient power according to TSA), while for ΔSOFA 72–96 h and vasopressor hours it is moderate: effects are consistent and statistically robust, but of small magnitude and without translation into length of stay or survival [10–13] (See Table 3). Component analyses confirm that the glucocorticoid explains vasopressor shortening and ICU day reduction in combined regimens, which does not modify certainty for survival when adding vitamin C or thiamine [20,34] (See Table 4).

**Table 3:** Summary of Findings (GRADE): HAT combination (hydrocortisone + vitamin C + thiamine) in adults with sepsis/septic shock.

Outcome	Anchor Estimator (95% CI, I²)	Certainty (GRADE)	Notes
28–30-day mortality	RR 1.02 (0.86–1.20); I² = 0% (Assouline 2021)	Low	Similar estimators in other SR with TSA (RR 0.96 (0.80–1.15), Na 2021; RR 1.03–1.05, Zayed 2022): no effect and, in TSA, insufficient power/futility.
ΔSOFA 72–96 h	WMD −0.82 (−1.15; −0.48); I² = 0% (Assouline 2021)	Moderate	Small but consistent benefit; positive TSA. Coherent in Wu 2021 (RCT: MD −0.86).
Duration of vasopressors	WMD −15 h (−25; −4) (Assouline 2021)	Moderate	Modest reduction reproduced in Na 2021 (−18.16 h) and Wu 2021 (−14.68 h).
ICU-LOS / Hospital-LOS	No differences	Low–Moderate	No changes in length of stay/ICU-free days.
Adverse events	Variable; tendency to hyperglycemia due to steroid	Low–Moderate	The GC component explains hemodynamic benefit (see Component-NMA).

GRADE reasons (HAT): • Risk of bias: Several open-label RCTs; some differences in day 3 SOFA management. • Inconsistency: Low for SOFA/vasopressors (I² = 0% in anchor); null for mortality (I² = 0%). • Imprecision: In mortality, TSA indicates optimal size not reached (futility/insufficiency conclusion). • Indirectness: Minimal; standard or hydrocortisone comparators. Abbreviations: AE = adverse events; CI = confidence interval; GRADE = Grading of Recommendations Assessment, Development and Evaluation; ICU-LOS = intensive care unit length of stay; MD = mean difference; RCT = randomized controlled trial; RR = risk ratio; SR = systematic review; TSA = Trial Sequential Analysis; WMD = weighted mean difference.

**Table 4:** Summary of Findings (GRADE): Vitamin C + thiamine (without corticosteroid) in adults with sepsis/septic shock.

Outcome	Anchor Estimator (95% CI, I²)	Certainty (GRADE)	Notes
28–30-day mortality / in-hospital	RR 1.02 (0.87–1.20); I² = 0% (Yao 2021, TSA)	Low	Confirmatory SR in Ge 2021 (in-hospital OR 1.11 (0.79–1.56); I² = 0%): no effect.
ΔSOFA 72–96 h	MD −0.63 (−0.96; −0.29); I² = 0% (Yao 2021)	Moderate	Small but stable effect; positive TSA.
Duration of vasopressors	MD −22.1 h (−30.5; −13.8) (Yao 2021) / WMD −17.7 h (−29.76; −4.98) (Ge 2021)	Moderate	Modest reduction reproduced in two SRs.
ICU-LOS / Hospital-LOS	No differences	Low–Moderate	Unchanged length of stay.
Adverse events	Limited data, no consistent signals	Low	Incomplete AE assessment in several RCTs.

GRADE reasons (Vit C + thiamine) • Risk of bias: Part of RCTs with incomplete blinding; moderate size. • Inconsistency: Low (I² = 0% in ΔSOFA), moderate in vasopressors between SRs. • Imprecision: Mortality with narrow CI around no-effect. • Indirectness: Minimal. Abbreviations: AE = adverse events; CI = confidence interval; GRADE = Grading of Recommendations Assessment, Development and Evaluation; ICU-LOS = intensive care unit length of stay; MD = mean difference; RCT = randomized controlled trial; RR = risk ratio; SR = systematic review; TSA = Trial Sequential Analysis; WMD = weighted mean difference.

## Discussion

### Main findings

IV vitamin C monotherapy showed a consistent signal of mortality benefit at 28–30 days when administered in sepsis (more than in shock), with intermediate doses and short courses; however, supportive outcomes were heterogeneous across reviews (reduction in vasopressor hours yes; ΔSOFA not reproducibly in pure RCTs). Conversely, combinations (HAT and vitamin C + thiamine) did not reduce mortality in reviews with the best methodological standards; their effect was limited to small and consistent improvements in ΔSOFA 72–96 h and vasopressor hours, without clear impact on length of stay or translation into survival.

Network/component meta-analysis confirmed that the corticosteroid is the main determinant of vasopressor shortening and slight ICU length reduction in combined regimens; adding vitamin C or thiamine did not modify survival in these schemes. Overall, the pattern is clear: monotherapy with probable short-term mortality benefit under specific conditions; combinations with modest hemodynamic benefit but no effect on mortality.

### Interpretation of results

Interest in adjuvants like IV vitamin C stems from a dual goal: reducing deaths and, if not possible, improving organ function/hemodynamic stability with safe interventions [1,3]. Vitamin C, water-soluble with a good safety profile, has biological plausibility (cofactor of dopamine-β-hydroxylase for catecholamines, NO modulation, endothelial antioxidation, and immunomodulation), which could reduce vasopressor support and, under favorable conditions, impact survival [4].

The most coherent signal appears with monotherapy, early, intermediate dose, and 3–4 days in sepsis, which aligns with the pathophysiological window [5,7,36,37]. In contrast, very high doses or longer exposures provided no advantages and, in sensitivity analyses, diluted the possible effect, suggesting a narrow therapeutic window [6,30]. It should be noted that the hypothesis of a beneficial effect under conditions of early initiation, intermediate dose, and short course is largely derived from post hoc subgroup aggregation across independent reviews, and was not pre-specified in the primary trials. As such, it should be interpreted as a hypothesis-generating finding rather than a confirmed clinical signal. The consistency of this pattern across methodologically diverse reviews lends it some plausibility, but prospective validation in trials designed around these specific parameters remains necessary before any clinical inference can be drawn. For HAT or vitamin C + thiamine, small decreases in ΔSOFA and vasopressor hours are plausible (steroid effect; metabolic role of thiamine), but are not accompanied by replicable improvements in metrics that matter to patients/hospitals; furthermore, component-NMA attributes the hemodynamic benefit to the corticosteroid, not the vitamins [10–13,20,34,41].

Moreover, the “multiplication” of reviews is partially justifiable (new and large RCTs, need to update monotherapy vs combinations, sepsis vs shock, dose/timing/duration) [10–12,20,34]. However, our CCA showed very high overlap: many reviews re-synthesize the same set of RCTs within each silo. Therefore, their incremental value depends on whether they add high AMSTAR-2 with well-reported protocol/excluded list/publication bias, prespecified and plausible subgroups (25–100 mg/kg/d vs > 100 mg/kg/d; initiation ≤24 h; 3–4 days; sepsis vs shock), TSA clarifying sufficiency/futility, or component-NMA assigning the effect to corticosteroid rather than vitamins [10–12,20,34].

It should be noted that high CCA does not invalidate the findings of individual reviews nor the conclusions drawn from them. Its primary implication is one of evidence redundancy: the proliferation of reviews in this field has not expanded the primary evidence base, as most analyses draw from the same compact set of RCTs. The consequence for certainty grading is indirect but relevant: because GRADE ratings are determined by the characteristics of underlying primary trials — not by the number of reviews analyzing them — additional reviews over the same RCTs do not increase certainty. High overlap therefore reinforces the rationale for the anchor estimator strategy and explains why certainty ratings remain low-to-moderate despite the large number of available reviews.

Regarding evaluated outcomes, surrogates serve as early signals (more sensitive than mortality) and for process monitoring, but their value for adoption decisions requires: (1) clinically relevant size (in SOFA, ≥ 1–2 points) and (2) consistent relationship with hard outcomes or resource use (length of stay, readmissions, mortality). Here, observed differences (SOFA around 0.6–0.9 points; vasopressors −15 to −22 h) are small, sensitive to co-interventions, and do not translate into length of stay or improved survival; moreover, the corticosteroid explains most of the combination effect [10–13,34]. For managers, introducing an intervention based solely on surrogates is not justifiable; they may guide hypotheses or stops for futility/benefit in RCTs with TSA, but do not replace clinical outcomes.

### Interpretation of certainty (GRADE)

Many SRs do not imply high certainty. With GRADE, certainty depends on underlying RCTs: clinical heterogeneity, imprecision from moderate sizes, and possible publication bias limit certainty of monotherapy on 28–30 d mortality to low-moderate [5–7]. In HAT and vitamin C + thiamine, mortality remains at low certainty (stable null effect or TSA with insufficiency/futility), while ΔSOFA and vasopressors reach moderate certainty due to consistency and robustness, but magnitudes are small and do not justify practice changes [10–13]. With a limited and overlapping core of RCTs, certainty is unlikely to increase without larger and better-designed trials.

### Implications for clinical practice

Our findings do not support routine use of HAT or vitamin C + thiamine to reduce mortality; observed hemodynamic benefits are small and attributable to the corticosteroid [20,34]. We do not recommend IV vitamin C monotherapy as standard; at most, a conditional/weak recommendation only in early sepsis, intermediate dose, 3–4 days, under protocol with audit and safety, preferably in RCTs [5–7,45]. This position does not contradict current guidelines, which advise against routine vitamin C [45].

### Limitations

The synthesis relies on published reviews; although we prioritized high AMSTAR-2 and documented very high overlap to avoid RCT double-counting, clinical heterogeneity, imprecision, and publication bias persist in some large meta-analyses. We did not perform de novo meta-analyses and thus depend on each review’s analytical decisions. An important methodological limitation is our approach of selecting a single anchor estimator for each outcome rather than performing formal synthesis of all available estimators. In contexts of very high CCA — as observed across all three silos in this review — averaging effect sizes from reviews that share the same primary RCTs would artificially inflate precision and violate the independence assumption underlying standard meta-analytic pooling. The anchor strategy was adopted to avoid this, prioritizing the most methodologically robust review per outcome and regimen. Nevertheless, this approach introduces subjectivity in anchor selection that could influence GRADE ratings. To address this, we present a sensitivity analysis in S4 Table, showing that substituting the primary anchor with the second- and third-ranked reviews by AMSTAR-2 score does not alter the direction or certainty category of any GRADE conclusion. The absence of prospective registration constitutes an additional limitation of this umbrella review, as it precludes independent verification that the protocol was not modified after data collection began. To partially mitigate this, we adhered to PRIOR reporting guidelines, documented all methodological decisions prior to analysis, and maintained an auditable extraction log with timestamps and author attribution, allowing critical evaluation and replication of our procedures. Finally, recent RCTs may not be included. However, given the consistent pattern and observed effect sizes, few isolated trials would substantially change the overall conclusions. We recommend periodic updates.

The high I² values observed for vasopressor duration (e.g., I² = 95% in Zhu 2022; I² = 45% in Ge 2021) and, to a lesser extent, for ΔSOFA, reflect sources of heterogeneity that go well beyond expected clinical variation. At least three operational factors contribute to this. First, the choice of first-line vasopressor differed across primary trials: norepinephrine was standard in most Western ICUs, whereas some Asian trials included dopamine or vasopressin as primary agents, directly affecting the baseline duration and dose-tapering trajectory. Second, the definition of “vasopressor duration” was not uniform: some trials recorded total hours of any vasopressor infusion, others reported time to cessation of the primary agent only, and at least one defined the endpoint as vasopressor-free days within a fixed window. Third, the timing and dose of vitamin C initiation varied considerably across trials included in each review, which interacts with vasopressor weaning in ways that are not linear. These distinctions are not merely semantic: pooling studies with different vasopressor definitions under a single MD or WMD produces a composite estimate of uncertain clinical meaning, which in part explains why vasopressor reduction was statistically significant in some SRs but not replicable in TSA-anchored analyses.

### Conclusions and recommendations

The available evidence does not support routine use of intravenous vitamin C in adults with sepsis or septic shock, either as monotherapy or in combinations (HAT or vitamin C plus thiamine). In combinations, no mortality reduction is observed. The described hemodynamic effects—modest decreases in ΔSOFA and vasopressor hours—are small, of limited clinical relevance, and primarily attributable to the corticosteroid component. Regarding monotherapy, although some high-quality systematic reviews point to a possible signal of mortality benefit under specific conditions (early administration, intermediate dose, short courses, and in sepsis more than shock), overall certainty remains low-to-moderate and is constrained by insufficient sample sizes, clinical heterogeneity, publication bias indicators, and very high overlap among reviews, which precludes firm clinical recommendations.

Consequently, intravenous vitamin C—either alone or in combination—is not recommended as part of routine management of sepsis or septic shock. The signal observed with monotherapy warrants, instead, multicenter and rigorous clinical trials exploring defined scenarios (initiation ≤24 h, intermediate dose for 3–4 days, explicit distinction between sepsis and shock, and consideration of baseline vitamin C status), with clinically relevant outcomes (28–30-day mortality), standardized risk of bias assessment, and sequential analysis when appropriate. Furthermore, future syntheses are required that reduce overlap, integrate larger RCTs, and apply formal and transparent publication bias assessments, aiming to decrease current uncertainty and clarify the true role of this intervention.

## Supporting information

S1 Table PRIOR statement—a reporting guideline for overviews of reviews. (DOCX)

S1 Checklist PRISMA 2020 checklist. (DOCX)

S2 Table Search strategy. (DOCX)

S3 Table Methodological Quality Assessment (AMSTAR-2). The table presents domain-level ratings for all 16 AMSTAR-2 items and the overall confidence rating for each review. Shaded headers denote AMSTAR-2 critical domains (items 2, 4, 7, 9, 11, 13, and 15).
(DOCX)

S4 Table Sensitivity Analysis: Stability of GRADE Conclusions Across Anchor Estimator Substitution (2nd and 3rd Ranked Reviews by AMSTAR-2 Score). Anchor selection criteria (applied a priori): (1) highest AMSTAR-2 overall rating; (2) strict PICO alignment with 28–30-day mortality horizon; (3) random-effects model declared as primary; (4) TSA availability; (5) pure RCT designs preferred over mixed RCT + observational. Within ties on AMSTAR-2, TSA availability was the deciding criterion. For the vitamin C + thiamine silo, only one SR was available; sensitivity analysis across alternate anchors was therefore not applicable. Abbreviations: RR = risk ratio; OR = odds ratio; CI = confidence interval; TSA = Trial Sequential Analysis; NMA = network meta-analysis; CINeMA = Confidence in Network Meta-Analysis; HAT = hydrocortisone + ascorbic acid + thiamine; GC = glucocorticoid; GRADE = Grading of Recommendations, Assessment, Development and Evaluations; SR = systematic review; NR = not reported.
(DOCX)

S5 Table Characteristics of included studies. (DOCX)
