# Supplementary material for: Monotherapy or combinations? Intravenous vitamin C in sepsis and septic shock: An umbrella review of 31 systematic reviews
Source: PLoS One. 2026 Jul 1;21(7):e0351072. doi: 10.1371/journal.pone.0351072 (PMC13322531; doi:10.1371/journal.pone.0351072)
Supplement: S1 Table — (DOCX) [file pone.0351072.s001.docx]

**Supplementary Table 1. PRIOR statement—a reporting guideline for overviews of reviews**

| **Section** | **Item No.** | **PRIOR Item** | **Compliant** | **Location (page/section)** |
| --- | --- | --- | --- | --- |
| TITLE | 1 | Identify the report as an overview of reviews. | Yes | Title (p. 1) |
| ABSTRACT | 2 | Provide a comprehensive and accurate summary of the purpose, methods, and results of the overview of reviews. | Yes | Abstract (p. 1–2) |
| INTRODUCTION | 3 | Describe the rationale for conducting the overview of reviews in the context of existing knowledge. | Yes | Introduction (p. 2–3) |
| INTRODUCTION | 4 | Provide an explicit statement of the objective(s) or question(s) addressed by the overview of reviews. | Yes | End of Introduction (p. 3) |
| METHODS | 5a | Specify inclusion and exclusion criteria for the overview of reviews. | Yes | Selection Criteria (p. 4–5) |
| METHODS | 5b | Specify the definition of 'systematic review' used in inclusion criteria. | Partial | Implicitly defined (p. 4) |
| METHODS | 6 | Specify all databases, registers, websites, and other sources searched, and date last searched. | Yes | Search Strategy (p. 3–4; Supplementary 1) |
| METHODS | 7 | Present full search strategies for all databases, registers, and websites. | Yes | Supplementary Material 1 |
| METHODS | 8a | Describe methods used to decide whether each review met inclusion criteria. | Yes | Selection Process (p. 5) |
| METHODS | 8b | Describe how overlap across systematic reviews was identified and managed. | Yes | Overlap Analysis (p. 8–9) |
| METHODS | 9a | Describe methods used to collect data from included reviews. | Yes | Data Extraction (p. 5–6) |
| METHODS | 9b | Describe how overlap at primary study level was managed. | Yes | Overlap Analysis and CCA formula (p. 8–9) |
| METHODS | 9c | Specify methods used to manage discrepant data across reviews. | Yes | Consensus resolution (p. 5–6) |
| METHODS | 10 | List and define all variables and outcomes for which data were sought. | Yes | Table Construction (p. 6–7) |
| METHODS | 11a | Describe methods used to assess risk of bias of included systematic reviews. | Yes | Risk of Bias (AMSTAR-2) (p. 7–8; Supplementary 3) |
| METHODS | 11b | Describe methods used to assess RoB of primary studies in the reviews. | Yes | Extracted as Cochrane RoB 2, Jadad, etc. (p. 5–6, 8) |
| METHODS | 11c | Describe methods used to assess RoB of any supplemental primary studies. | N/A | Not applicable – no supplemental studies included |
| METHODS | 12a | Describe synthesis methods and rationale for choices. | Yes | Statistical Analysis (p. 9) |
| METHODS | 12b | Describe methods used to explore heterogeneity. | Yes | I², TSA, subgroup analyses (p. 7–9) |
| METHODS | 12c | Describe sensitivity analyses to assess robustness. | Yes | Mentioned in results and GRADE (p. 8, 12) |
| METHODS | 13 | Describe methods used to assess reporting bias. | Yes | Publication bias via funnel/Egger (p. 7–8, Tables 2–4) |
| METHODS | 14 | Describe methods used to assess certainty/confidence (GRADE). | Yes | Certainty of Evidence (GRADE) (p. 8; Tables 2–4) |
| RESULTS | 15a | Describe results of search and selection process with flow diagram. | Yes | Study Selection and Supplementary 2 (p. 9) |
| RESULTS | 15b | Provide list of excluded studies with reasons. | Yes | Full-text exclusions listed (p. Supplementary 2) |
| RESULTS | 16 | Cite each included review and present characteristics. | Yes | Table 1 (p. 9–10) |
| RESULTS | 17 | Describe extent of primary study overlap. | Yes | Overlap Index (CCA) (p. 8–9) |
| RESULTS | 18a | Present risk of bias assessment for each review. | Yes | AMSTAR-2 results (p. 9–10; Supplementary 3) |
| RESULTS | 18b | Present RoB of primary studies from reviews. | Yes | Reported in Risk of Bias section (p. 7–8) |
| RESULTS | 18c | Present RoB of supplemental primary studies. | N/A | Not applicable |
| RESULTS | 19a | Summarize evidence from all included reviews for each outcome. | Yes | Results and Tables 2–4 (p. 10–13) |
| RESULTS | 19b | Present results of heterogeneity analyses. | Yes | GRADE rationale and Discussion (p. 12–13) |
| RESULTS | 19c | Present sensitivity analyses. | Yes | Mentioned in text (p. 8, 12) |
| RESULTS | 20 | Present assessment of reporting bias in results. | Yes | GRADE and AMSTAR (p. 7–8, 12) |
| RESULTS | 21 | Present certainty/confidence in evidence for each outcome. | Yes | GRADE SoF Tables (p. 11–13) |
| DISCUSSION | 22a | Summarize main findings including discrepancies. | Yes | Main Findings (p. 13–14) |
| DISCUSSION | 22b | Interpret results in context of other evidence. | Yes | Interpretation of Results (p. 14–15) |
| DISCUSSION | 22c | Discuss limitations of evidence and overview methods. | Yes | Limitations (p. 17) |
| DISCUSSION | 22d | Discuss implications for practice, policy, and future research. | Yes | Implications & Conclusions (p. 16–18) |
| OTHER INFO | 23a | Provide registration info or state unregistered. | No | Explicitly states not registered (p. 17) |
| OTHER INFO | 23b | Indicate where protocol can be accessed or note absence. | Yes | Follows PRIOR and Aromataris; not registered (p. 3–4, 17) |
| OTHER INFO | 23c | Describe amendments to protocol. | N/A | Not applicable |
| OTHER INFO | 24 | Describe funding sources and sponsor roles. | Yes | Funding section (p. 18) |
| OTHER INFO | 25 | Declare any competing interests. | Yes | Conflict of Interest (p. 18) |
| OTHER INFO | 26a | Provide corresponding author contact. | Yes | First page (p. 1) |
| OTHER INFO | 26b | Describe author contributions and guarantor. | Yes | Authors’ contribution (p. 18–19) |
| OTHER INFO | 27 | Report availability of data, forms, code, and materials. | Partial | Data available upon request; no code (p. 18) |
