# Supplementary material for: Monotherapy or combinations? Intravenous vitamin C in sepsis and septic shock: An umbrella review of 31 systematic reviews
Source: PLoS One. 2026 Jul 1;21(7):e0351072. doi: 10.1371/journal.pone.0351072 (PMC13322531; doi:10.1371/journal.pone.0351072)
Supplement: S2 Table — (DOCX) [file pone.0351072.s003.docx]

**Supplementary Table 2. Search strategy**

| Search strategy in PUBMED | |
| --- | --- |
| #1 | ("Sepsis"[Mesh] OR "Shock, Septic"[Mesh] OR sepsis[tiab] OR "septic shock"[tiab] OR "severe sepsis"[tiab]) |
| #2 | (("Ascorbic Acid"[Mesh] OR "ascorbic acid"[tiab] OR "vitamin C"[tiab] OR ascorbate[tiab] OR "ascorbic"[tiab]) AND (intraven*[tiab] OR IV[tiab] OR infusion*[tiab] OR parenteral*[tiab]) OR ((hydrocortisone[tiab] OR "Hydrocortisone"[Mesh]) AND (thiamine[tiab] OR "vitamin B1"[tiab] OR "Thiamine"[Mesh] OR aneurine[tiab]) AND ("ascorbic acid"[tiab] OR "vitamin C"[tiab] OR ascorbate[tiab])) OR (("ascorbic acid"[tiab] OR "vitamin C"[tiab] OR ascorbate[tiab]) AND (thiamine[tiab] OR "vitamin B1"[tiab] OR aneurine[tiab]) AND NOT hydrocortisone[tiab]) OR ("metabolic resuscitation"[tiab] OR "Marik bundle"[tiab] OR "hydrocortisone-ascorbic-thiamine"[tiab])) |
| #3 | "systematic review"[tiab] OR "systematic reviews"[tiab] OR "meta-analysis"[tiab] OR "meta-analyses"[tiab] OR "meta analysis"[tiab] OR "metaanalysis"[tiab] OR "revisión sistemática"[tiab] OR "revisiones sistemáticas"[tiab] OR "metaanálisis"[tiab] OR "meta análisis"[tiab] OR "overview of reviews"[tiab] OR "umbrella review"[tiab] OR "umbrella reviews"[tiab] OR "review of reviews"[tiab] OR "scoping review"[tiab] OR "narrative review"[tiab] OR "pooled analysis"[tiab] |
| #4 | #1 AND #2 AND #3 |
| Search strategy in SCOPUS | |
| #1 | TITLE-ABS-KEY ( sepsis OR "septic shock" OR "severe sepsis" ) |
| #2 | TITLE-ABS-KEY ( ( "vitamin c" OR ascorbate OR "ascorbic acid" OR ascorbic ) AND ( intraven* OR infusion* OR IV OR parenteral* ) OR ( hydrocortisone W/3 thiamine W/3 ( "vitamin c" OR ascorbate OR "ascorbic acid" ) ) OR ( ( "vitamin c" OR ascorbate OR "ascorbic acid" ) W/3 thiamine AND NOT hydrocortisone ) OR ( "metabolic resuscitation" OR "Marik bundle" ) ) |
| #3 | TITLE-ABS-KEY ("systematic review" OR "systematic reviews" OR "meta-analysis" OR "meta analysis" OR "metaanalysis" OR "umbrella review" OR "overview of reviews" OR "review of reviews" OR "scoping review" OR "narrative review" OR "pooled analysis") |
| #4 | #1 AND #2 AND #3 |
| Search strategy in Web of Science | |
| #1 | TS=(sepsis OR "septic shock" OR "severe sepsis") |
| #2 | TS=(("vitamin c" OR ascorbate OR "ascorbic acid" OR ascorbic) NEAR/3 (intraven* OR infusion* OR IV OR parenteral*) OR (hydrocortisone NEAR/3 thiamine NEAR/3 ("vitamin c" OR ascorbate OR "ascorbic acid")) OR (("vitamin c" OR ascorbate OR "ascorbic acid") NEAR/3 thiamine NOT hydrocortisone) OR ("metabolic resuscitation" OR "Marik bundle")) |
| #3 | TS=("systematic review" OR "systematic reviews" OR "meta-analysis" OR "meta analysis" OR "umbrella review" OR "overview of reviews" OR "review of reviews" OR "scoping review" OR "narrative review") |
| #4 | #1 AND #2 AND #3 |
| Search strategy in EMBASE | |
| #1 | ('ascorbic acid'/exp OR 'acidum ascorbicum' OR 'acidylina' OR 'adenex' OR 'afj c' OR 'agrumina' OR 'allercorb' OR 'allescorb' OR 'antiscorbutic vitamin' OR 'arcavit c' OR 'arcavite c' OR 'arkovital c' OR 'ascelat' OR 'ascofar' OR 'ascomed' OR 'asconvita' OR 'ascor' OR 'ascor l 500' OR 'ascorbate' OR 'ascorbate sodium' OR 'ascorbic acid' OR 'ascorbic acid potassium salt' OR 'ascorbicap' OR 'ascorbicin' OR 'ascorbico' OR 'ascorbin' OR 'ascorbina' OR 'ascorbinic acid' OR 'ascorbit' OR 'ascorbite' OR 'ascorbitol' OR 'ascorbivit' OR 'ascorbivite' OR 'ascorbone' OR 'ascorbutina' OR 'ascorbyl' OR 'ascorbyn' OR 'ascorcee' OR 'ascorgil' OR 'ascorin' OR 'ascormin' OR 'ascorteal' OR 'ascorval' OR 'ascorvel' OR 'ascorvit' OR 'ascorvite' OR 'ascorvitina' OR 'askorbin' OR 'austrovit c' OR 'austrovite c' OR 'bentavit c' OR 'bentavite c' OR 'c crivit' OR 'c ine' OR 'c level' OR 'c lisa' OR 'c long' OR 'c monovit' OR 'c monovite' OR 'c prana' OR 'c rivitin' OR 'c rivitine' OR 'c sol' OR 'c tamin' OR 'c tamine' OR 'c tonic' OR 'c tron' OR 'c vescent' OR 'c vicotrat' OR 'c vicotrate' OR 'c vimin' OR 'c vimine' OR 'c vit' OR 'c vita' OR 'c vital' OR 'c vitam' OR 'c vite' OR 'c-tard' OR 'c-vimin' OR 'c-will' OR 'cantan' OR 'cantaxin' OR 'catavin c' OR 'ce arom' OR 'ce limo' OR 'ce major' OR 'ce quin' OR 'ce quine' OR 'ce vi sol' OR 'ce vita' OR 'ce vitan' OR 'ce-vi-sol' OR 'cebetate' OR 'cebicure' OR 'cebiolon' OR 'cebion' OR 'cebione' OR 'cecap' OR 'cecon' OR 'cecon drops' OR 'cecon solution' OR 'cecone' OR 'cecorbin' OR 'cecorbine' OR 'cecorbyl' OR 'cecorbyle' OR 'cecrisina' OR 'cedon' OR 'cedone' OR 'cedoxon' OR 'cedoxone' OR 'cee-500' OR 'ceevifil' OR 'cegiolan' OR 'celaskon' OR 'celaskone' OR 'celin' OR 'celine' OR 'cenetone' OR 'cenol' OR 'cenolate' OR 'cequinyl' OR 'cereon' OR 'cergona' OR 'cescorbat' OR 'cetamican' OR 'cetamid' OR 'cetamin knoll australia' OR 'cetamine' OR 'cetamine knoll australia' OR 'cetebe' OR 'ceterapion' OR 'ceterapione' OR 'cetrinets' OR 'cevalin' OR 'cevaline' OR 'cevatine' OR 'cevex' OR 'cevibram' OR 'cevigal' OR 'cevigen' OR 'cevigol' OR 'cevikap' OR 'cevilat' OR 'cevimin' OR 'cevimine' OR 'cevisol' OR 'cevit' OR 'cevita' OR 'cevitamic acid' OR 'cevitamin' OR 'cevitaminic acid' OR 'cevitaminum kolin' OR 'cevitan' OR 'cevite' OR 'cevitex' OR 'cevitil' OR 'cevitol' OR 'cewin' OR 'chewcee' OR 'chivibit c' OR 'ci drol' OR 'ciamin' OR 'ciamina ormo' OR 'ciergin' OR 'cifilina' OR 'cipca' OR 'cisir' OR 'citamino' OR 'cith' OR 'citoascorbina' OR 'citoxyl' OR 'citran' OR 'citravite' OR 'citritabs' OR 'citrovitamina' OR 'civigor' OR 'civitin' OR 'civitine' OR 'co biagini' OR 'concemin' OR 'cortalex' OR 'd ascorbic acid' OR 'd xyloascorbic acid' OR 'dagrascorbin' OR 'dagravit c' OR 'dancimin c' OR 'davitamon c' OR 'dayvital' OR 'delo c' OR 'difvitamin c' OR 'dropice' OR 'dumovit c' OR 'dumovite c' OR 'e ascorbic acid' OR 'e xyloascorbic acid' OR 'erftamin c' OR 'erftamine c' OR 'esuron' OR 'esurvit' OR 'esurvite' OR 'flavettes' OR 'godabion c' OR 'gregovite c' OR 'hicee' OR 'hybrin' OR 'ido c' OR 'ikacee' OR 'inovitan c' OR 'irocevit' OR 'irocevite' OR 'jarexin' OR 'jarexine' OR 'l 3 keto hexuronic acid lactone' OR 'l ascorbic acid' OR 'l xylo ascorbic acid' OR 'l xyloascorbic acid' OR 'lacivit' OR 'lacivite' OR 'laroscorbine' OR 'leder c' OR 'leder-c' OR 'lemascorb' OR 'levo ascorbate' OR 'levo ascorbic acid' OR 'limcee' OR 'limo ce' OR 'liqui cee' OR 'mega-c/a plus' OR 'myascorbin' OR 'natrascorb' OR 'novo ascorbic' OR 'nybadol' OR 'paa 500' OR 'parkovit c' OR 'pascorbin' OR 'pharmascorbine' OR 'pharmatovit c' OR 'pharmatovite c' OR 'planavit c' OR 'planavite c' OR 'plivit c' OR 'plivite c' OR 'potassium ascorbate' OR 'pro-c' OR 'proscorbin' OR 'proscorbine' OR 'redoxon' OR 'redoxon c' OR 'redoxon forte' OR 'ribena' OR 'scorbacid' OR 'scorbacide' OR 'scorbettes' OR 'scorbex' OR 'scorbin c' OR 'scorbitol' OR 'scorbumine' OR 'scottavit c' OR 'scottavite c' OR 'secorbate' OR 'sevalin' OR 'sigmavit c' OR 'sigmavite c' OR 'sodascorbate' OR 'sodium ascorbate' OR 'sodium l ascorbate' OR 'sweetcee' OR 'synum c' OR 'take-c' OR 'tanvimil-c' OR 'testascorbic' OR 'ucemine c' OR 'upsa c' OR 'upsa-c' OR 'upsavit' OR 'vagi-c' OR 'vi ci sin' OR 'vi dom c' OR 'vi-c 500' OR 'vicef' OR 'vicelat' OR 'vicetrin' OR 'vici monico' OR 'viciman' OR 'vicin' OR 'vicitina' OR 'vicon' OR 'viforcit' OR 'viforcite' OR 'viscorin' OR 'viscorine' OR 'vita-cedol orange' OR 'vita-gem c' OR 'vitac' OR 'vitace' OR 'vitacee' OR 'vitaci' OR 'vitacimin' OR 'vitacimine' OR 'vitacin' OR 'vitacine' OR 'vitamin c' OR 'vitamin c 1' OR 'vitaplex c' OR 'vitapric' OR 'vitapur c' OR 'vitasan c' OR 'vitascorbin' OR 'vitascorbine' OR 'vitascorbol' OR 'vitascorbol 500' OR 'vitelix c' OR 'vitocee' OR 'vorange' OR 'wandervit c' OR 'wandervite c' OR 'witamina c' OR 'xitix' OR 'xon-ce' OR 'xyloascorbic acid') |
| #2 | ('sepsis'/exp OR 'abdominal sepsis' OR 'focal sepsis' OR 'intraabdominal sepsis' OR 'sepsis' OR 'sepsis syndrome' OR 'septic disease') |
| #3 | 'systematic review'/exp OR 'meta analysis'/exp OR 'review'/exp OR 'systematic review':ti,ab,kw OR 'systematic reviews':ti,ab,kw OR 'meta-analysis':ti,ab,kw OR 'meta analysis':ti,ab,kw OR 'metaanalysis':ti,ab,kw OR 'umbrella review':ti,ab,kw OR 'overview of reviews':ti,ab,kw OR 'review of reviews':ti,ab,kw OR 'scoping review':ti,ab,kw OR 'narrative review':ti,ab,kw OR 'pooled analysis':ti,ab,kw |
| #4 | #1 AND #2 AND #3 |
