# Supplementary material for: Monotherapy or combinations? Intravenous vitamin C in sepsis and septic shock: An umbrella review of 31 systematic reviews
Source: PLoS One. 2026 Jul 1;21(7):e0351072. doi: 10.1371/journal.pone.0351072 (PMC13322531; doi:10.1371/journal.pone.0351072)
Supplement: S3 Table — The table presents domain-level ratings for all 16 AMSTAR-2 items and the overall confidence rating for each review. Shaded headers denote AMSTAR-2 critical domains (items 2, 4, 7, 9, 11, 13, and 15). (DOCX) [file pone.0351072.s004.docx]

**Supplementary Table 3: Methodological Quality Assessment (AMSTAR-2)**

| First author and year | **1** | **2** | **3** | **4** | **5** | **6** | **7** | **8** | **9** | **10** | **11** | **12** | **13** | **14** | **15** | **16** | **Overall** |
| --- | --- | --- | --- | --- | --- | --- | --- | --- | --- | --- | --- | --- | --- | --- | --- | --- | --- |
| Zeng 2023 | Yes | Not rep. | Yes | Yes | Yes | Yes | Partial | Yes | Yes | Not rep. | Yes | Partial | Yes | Yes | Yes | Yes | Moderate |
| Luo 2023 | Yes | Yes | Yes | Yes | Yes | Yes | Partial | Yes | Yes | Not rep. | Yes | Partial | Yes | Partial | Partial | Yes | Moderate |
| Liang 2023 | Yes | Yes | Yes | Yes | Yes | Yes | Yes | Yes | Yes | Not rep. | Yes | Yes | Yes | Yes | Yes (Egger) | Yes | High |
| Kato 2023 | Yes | Not rep. | Yes | Yes | Yes | Yes | Partial | Yes | Yes | Not rep. | Yes | Partial | Yes | Partial | Partial | Yes | Moderate |
| Hung 2023 | Yes | Yes | Yes | Yes | Yes | Yes | Yes | Yes | Yes | Not rep. | Yes | Yes | Yes | Yes | Yes | Yes | High |
| Lu & Mao 2023 | Yes | Yes | Yes | Yes | Yes | Yes | Yes | Yes | Yes | Not rep. | Yes | Yes | Yes | Yes | Yes | Yes | High |
| Liang 2023 | Yes | Yes | Yes | Yes | Yes | Yes | Yes | Yes | Yes | Not rep. | Yes | Yes | Yes | Yes | Yes (Egger) | Yes | High |
| Lee 2023 | Yes | Yes | Yes | Yes | Yes | Yes | Yes | Yes | Yes | Not rep. | Yes | Yes | Yes | Yes | Yes | Yes | High |
| Wen 2023 | Yes | Yes | Yes | Yes | Yes | Yes | Partial | Yes | Yes | Not rep. | Yes | Partial | Yes | Yes | Yes (Egger+) | Yes | Moderate |
| Brown 2022 | Yes | Not rep. | Yes | Yes | Partial | Partial | Not rep. | Yes | Yes | Not rep. | Yes | Not rep. | Partial | Yes | Yes (funnel) | Yes | Moderate |
| Chen 2022 | Yes | Yes | Yes | Yes | Yes | Yes | Yes | Yes | Yes | Not rep. | Yes | Yes | Yes | Yes | Partial | Yes | High |
| Tariq 2022 | Yes | Not rep. | Yes | Yes | Yes | Yes | Partial | Yes | Yes | Not rep. | Yes | Partial | Yes | Partial | Partial | Yes | Moderate |
| Muhammad 2022 | Yes | Yes | Yes | Yes | Yes | Yes | Yes | Yes | Yes | Not rep. | Yes | Yes | Yes | Yes | Partial | Yes | High |
| Zhu 2022 | Yes | Yes | Yes | Yes | Yes | Yes | Partial | Yes | Yes | Not rep. | Yes | Partial | Yes | Partial | Partial | Yes | Moderate |
| Martimbianco 2022 | Yes | Yes | Yes | Yes | Yes | Yes | Yes | Yes | Yes | Not rep. | Yes | Yes | Yes | Yes | Partial | Yes | High |
| Cai 2022 | Yes | Not rep. | Yes | Yes | Yes | Yes | Not rep. | Yes | Yes | Not rep. | Yes | Partial | Yes | Yes | Funnel | Yes | Moderate |
| Na 2021 | Yes | Yes | Yes | Yes | Yes | Yes | Yes | Yes | Yes | Not rep. | Yes | Yes | Yes | Yes | Funnel | Yes | High |
| Fujii 2022 | Yes | Yes | Yes | Yes | Yes | Yes | Yes | Yes | Yes | Not rep. | Yes (NMA) | Yes (CINeMA) | Yes | Yes (coherencia) | Yes | Yes | High |
| Assouline 2021 | Yes | Yes | Yes | Yes | Yes | Yes | Yes | Yes | Yes | Not rep. | Yes | Yes | Yes | Yes | Yes | Yes | High |
| Wu 2021 | Yes | Yes | Yes | Yes | Yes | Yes | Partial | Yes | Yes | Not rep. | Yes | Partial | Yes | Partial | Funnel | Yes | High |
| Scholz 2021 | Yes | Yes | Yes | Yes | Yes | Yes | Partial | Yes | Yes | Not rep. | Yes | Partial | Yes | Yes | Yes | Yes | High |
| Li 2021 | Yes | Yes | Yes | Yes | Yes | Yes | Partial | Yes | Yes | Not rep. | Yes | Yes | Yes | Partial | Funnel | Yes | High |
| Kanchanasurakit 2021 | Yes | Not rep. | Yes | Yes | Yes | Yes | Partial | Yes | Yes | Not rep. | Yes | Partial | Yes | Partial | Funnel | Yes | Moderate |
| Fong 2021 | Yes | Yes | Yes | Yes | Yes | Yes | Yes | Yes | Yes | Not rep. | Yes (NMA) | Yes (CINeMA) | Yes | Yes (coherencia) | Yes | Yes | High |
| Somagutta 2021 | Yes | Not rep. | Yes | Yes | Yes | Yes | Partial | Yes | Yes | Not rep. | Yes | Partial | Yes | Yes | Funnel | Yes | Moderate |
| Feng 2021 | Yes | Not rep. | Yes | Yes | Yes | Yes | Partial | Yes | Yes | Not rep. | Yes | Partial | Yes | Partial | Funnel (visual) | Yes | Moderate |
| Ge 2021 | Yes | N. | Yes | Yes | Yes | Yes | Partial | Yes | Yes | Not rep. | Yes | Partial | Yes | Partial | Not rep. | Yes | Moderate |
| Zayed 2022 | Yes | Yes | Yes | Yes | Yes | Yes | Yes | Yes | Yes | Not rep. | Yes | Yes | Yes | Yes | Funnel | Yes | High |
| Qian 2020 | Yes | Not rep. | Yes | Yes | Yes | Yes | Partial | Yes | Yes | Not rep. | Yes | Partial | Yes | Partial | Not rep. | Yes | Moderate |
| Shi & Tie 2020 | Yes | Not rep. | Partial | Partial | Not rep. | Not rep. | Not rep. | Yes | Partial | Not rep. | Partial | Not rep. | Partial | Partial | Not rep. | Yes | Low |
| Wei 2020 | Yes | Not rep. | Yes | Yes | Yes | Partial | Not rep. | Yes | Yes | Not rep. | Yes | Partial | Yes | Partial | Funnel | Yes | Moderate |

The table presents domain-level ratings for all 16 AMSTAR-2 items and the overall confidence rating for each review. Shaded headers denote AMSTAR-2 critical domains (items 2, 4, 7, 9, 11, 13, and 15).
