# Supplementary material for: Monotherapy or combinations? Intravenous vitamin C in sepsis and septic shock: An umbrella review of 31 systematic reviews
Source: PLoS One. 2026 Jul 1;21(7):e0351072. doi: 10.1371/journal.pone.0351072 (PMC13322531; doi:10.1371/journal.pone.0351072)
Supplement: S4 Table — Anchor selection criteria (applied a priori): (1) highest AMSTAR-2 overall rating; (2) strict PICO alignment with 28–30-day mortality horizon; (3) random-effects model declared as primary; (4) TSA availability; (5) pure RCT designs preferred over mixed RCT + observational. Within ties on AMSTAR-2, TSA availability was the deciding criterion. For the vitamin C + thiamine silo, only one SR was available; sensitivity analysis across alternate anchors was therefore not applicable. Abbreviations: RR = risk ratio; OR = odds ratio; CI = confidence interval; TSA = Trial Sequential Analysis; NMA = network meta-analysis; CINeMA = Confidence in Network Meta-Analysis; HAT = hydrocortisone + ascorbic acid + thiamine; GC = glucocorticoid; GRADE = Grading of Recommendations, Assessment, Development and Evaluations; SR = systematic review; NR = not reported. (DOCX) [file pone.0351072.s005.docx]

**Supplementary Material 4.** **Sensitivity Analysis: Stability of GRADE Conclusions Across Anchor Estimator Substitution (2nd and 3rd Ranked Reviews by AMSTAR-2 Score)**

| **Silo** | **Anchor rank** | **Review (first author, year)** | **Estimator (95% CI)** | **I²** | **AMSTAR-2** | **GRADE certainty** | **GRADE conclusion** |
| --- | --- | --- | --- | --- | --- | --- | --- |
| **IV VITAMIN C MONOTHERAPY — 28-30 DAY MORTALITY** | | | | | | | |
| **IV Vit C Monotherapy** | **1st (primary anchor)** | **Hung 2023** | RR 0.76 (0.60–0.97) | NR | High | Low-moderate | Possible mortality benefit signal; certainty limited by imprecision, heterogeneity, and publication bias |
|  | 2nd | Lee 2023 | RR 0.73 (0.60–0.89); 28d: RR 0.71 (0.53–0.95) | NR | High | Low-moderate | Same direction and certainty category as primary anchor |
|  | 3rd | Martimbianco 2022 | RR 0.60 (0.45–0.80) | 0% | High | Low-moderate | Same direction and certainty category as primary anchor |
| **HAT (HYDROCORTISONE + VITAMIN C + THIAMINE) — 28-30 DAY MORTALITY** | | | | | | | |
| **HAT** | **1st (primary anchor)** | **Assouline 2021** | RR 1.02 (0.86–1.20) | 0% | High | Low | No mortality benefit; TSA indicates futility/insufficiency |
|  | 2nd | Na 2021 | RR 0.96 (0.80–1.15) | 0% | High | Low | Same direction and certainty category as primary anchor |
|  | 3rd | Zayed 2022 | RR 1.05 (0.85–1.30) | NR | High | Low | Same direction and certainty category as primary anchor |
| **VITAMIN C + THIAMINE (WITHOUT CORTICOSTEROID) — 28-30 DAY MORTALITY** | | | | | | | |
| **Vit C + Thiamine** | **1st (primary anchor; only available SR)** | **Ge 2021** | OR 1.11 (0.79–1.56) | 0% | Moderate | Low | No mortality benefit; single SR limits sensitivity analysis for this silo |
| **COMPONENT / NETWORK META-ANALYSIS — MORTALITY AND VASOPRESSOR HOURS** | | | | | | | |
| **NMA / Component** | **1st (primary anchor)** | **Fujii 2022** | No mortality benefit attributable to vitamin C or thiamine (all comparisons) | CINeMA | High | Low-moderate | Glucocorticoid component explains vasopressor reduction; no vitamin C/thiamine mortality effect |
|  | 2nd | Fong 2021 | Ascorbate RR 0.74 (0.57–0.97) (<90d); GC+fludro RR 0.89 (0.80–0.99) | CINeMA | High | Low-moderate | Direction consistent; glucocorticoid remains main driver. Certainty category unchanged |

Anchor selection criteria (applied a priori): (1) highest AMSTAR-2 overall rating; (2) strict PICO alignment with 28-30-day mortality horizon; (3) random-effects model declared as primary; (4) TSA availability; (5) pure RCT designs preferred over mixed RCT+observational. Within ties on AMSTAR-2, TSA availability was the deciding criterion. For the vitamin C + thiamine silo, only one SR was available; sensitivity analysis across alternate anchors was therefore not applicable. Abbreviations: RR = risk ratio; OR = odds ratio; CI = confidence interval; TSA = Trial Sequential Analysis; NMA = network meta-analysis; CINeMA = Confidence in Network Meta-Analysis; HAT = hydrocortisone + ascorbic acid + thiamine; GC = glucocorticoid; GRADE = Grading of Recommendations, Assessment, Development and Evaluations; SR = systematic review; NR = not reported.
