# Supplementary material for: Monotherapy or combinations? Intravenous vitamin C in sepsis and septic shock: An umbrella review of 31 systematic reviews
Source: PLoS One. 2026 Jul 1;21(7):e0351072. doi: 10.1371/journal.pone.0351072 (PMC13322531; doi:10.1371/journal.pone.0351072)
Supplement: S5 Table — (DOCX) [file pone.0351072.s006.docx]

**Supplementary material 5. Characteristics of included studies**

| **First author and year** | **Regimen (focus)** | **Population** | **Search period** | **Included designs** | **RCTs only** | **Total n** | **Mortality 28-30 d (effect, 95% CI, model/I²)** | **ΔSOFA 72-96 h (effect, 95% CI, model/I²)** | **Vasopressors (h) (effect, 95% CI, model/I²)** | **Registry** | **RoB primaries** | **Publ. bias** | **Method** |
| --- | --- | --- | --- | --- | --- | --- | --- | --- | --- | --- | --- | --- | --- |
| Zeng 2023 | IV Vit C monotherapy (high dose) | Adults (sepsis ± shock) | NR (in PDF) | RCT | 8 | 1,394 | Sepsis: RR 0.61 (0.46-0.82), I² = 0%; Shock: RR 0.76 (0.46-1.27), I² = 67% | MD -1.20 (-4.35; 1.94) → ns, I² = 90% | Sepsis: SMD -0.59 (-0.99; -0.19), I² = 27% | NR | Cochrane RoB 2 | Funnel/Egger | Random-effects MA |
| Luo 2023 | IV Vit C (sepsis RCTs) | Adults (sepsis ± shock) | NR | RCT | 10 | 1,426 | OR 0.61 (0.37-1.01), p = 0.05 (random) | MD -0.85 (-2.38; 0.67) → ns | MD -14.36 (-26.11; -2.61) | NR | Cochrane RoB 2 | Partial | Random-effects MA |
| Liang 2023 | IV Vit C (RCT; IVVC) + TSA | Adults (sepsis ± shock) | Up to Nov-28-2022 | RCT | 22 | 3,570 | RR 0.92 (0.81-1.04), I² = 26% (overall); monotherapy: RR 0.69 (0.52-0.93), I² = 57% | MD 0.04 (-0.55; 0.63) → ns, I² = 96% | MD -8.45 (-15.43; -1.47) | INPLASY | Cochrane RoB 2 | Egger | Random-effects MA + TSA |
| Kato 2023 | HAT | Adults (sepsis ± shock) | 1940-2022 | RCT | 9 | NR | OR 0.88 (0.56-1.39) → ns | MD -0.04 (-1.31; 1.22) → ns | MD -19.51 (-26.13; -12.88) | NR | Cochrane RoB 2 | NR | Random-effects MA |
| Hung 2023 | IV Vit C monotherapy + TSA | Critical (≈62-63% sepsis) | NR | RCT | 12 | 1,712 | RR 0.76 (0.60-0.97) (random) | MD -0.72 (ns) | MD -37.75 (-70.77; -4.73) | PROSPERO | Cochrane RoB 2 | Funnel/Egger | Random-effects MA + TSA |
| Lu & Mao 2023 | HAT + TSA | Adults (sepsis ± shock) | Up to Oct-31-2022 | RCT | 8 | 1,572 | RR 0.96 (0.83-1.11) → ns | MD -0.69 (-1.44; 0.06) → ns | MD -7.59 (-23.4; 8.23) → ns | NR | Cochrane RoB 2 | Funnel | Random-effects MA + TSA |
| Liang 2023 | IV Vit C (RCT) + TSA | Adults (sepsis ± shock) | Up to Jan-16-2023 | RCT | 18 | 3,364 | OR 0.89 (0.77-1.04) (overall); 25-100 mg/kg/d: OR 0.80 (0.65-0.97) | MD -0.62 (-1.00; -0.25) | MD -15.07 (-21.59; -8.55) | PROSPERO | Cochrane RoB 2 | Egger | Random-effects MA + TSA |
| Lee 2023 | IV Vit C monotherapy + TSA | Critical (≈62% sepsis) | NR | RCT | 16 | 2,130 | RR 0.73 (0.60-0.89) (overall); 28 d RR 0.71 (0.53-0.95) | MD -0.82 (-1.77; 0.14) → ns | MD -0.79 d (-1.24; -0.34) | PROSPERO | Cochrane RoB 2 | Funnel/Egger | Random-effects MA + TSA |
| Wen 2023 | "Vit C-containing therapy" (RCT) | Adults (sepsis ± shock) | Nov-23 | RCT | 24 | 3,759 | OR 0.86-0.87 (0.71-1.08) → ns | SMD 0.26 (0.09-0.42) | SMD -0.72 (-1.00; -0.44) | PROSPERO | Cochrane RoB 2 | Egger positive | Random-effects MA |
| Brown 2022 | Vit C (RCT + observational) | Adults (sepsis ± shock, some trauma/burns) | Up to 2021 | RCT + Obs | 11 | 4,078 | OR 0.92 (0.78-1.09) → ns | — | — | PROSPERO | Cochrane/NOS | Funnel asymmetric | Random-effects MA |
| Chen 2022 | "Vit C-containing therapy" (RCT) | Adults (sepsis ± shock) | NR | RCT | 16 | 2,985 | Overall 28 d OR 0.87 (0.71-1.08); Shock OR 1.09 (0.89-1.34); ICU OR 1.03 (0.84-1.25); In-hosp OR 1.06 (0.85-1.13); 90 d OR 1.23 (0.75-2.02) → all ns | — | — | PROSPERO | Cochrane RoB 2 | Partial | Random-effects MA |
| Tariq 2022 | High dose Vit C (RCT; mono/HAT) | Adults (sepsis/shock) | NR | RCT | 14 | 2,793 | ICU OR 0.98 (0.74-1.30); 30 d OR 0.87 (0.69-1.09); 90 d OR 1.10 (0.91-1.34) → ns | HAT WMD -0.52 (-1.01; -0.03); Monotherapy: ns | — | NR | Cochrane RoB 2 | NR | Random-effects MA |
| Muhammad 2022 | "Vit C-containing" (RCT; overall) | Adults (sepsis) | Up to Jan-2022 | RCT | 23 | 2,712 | OR 0.778 (0.635-0.954); p = 0.016 | MD -0.749 (-1.115; -0.383) | MD -1.034 d (-1.622; -0.445) | (GRADE approach) | Cochrane RoB 2 | Funnel | Random-effects MA |
| Zhu 2022 | IV Vit C monotherapy (RCT) | Adults (sepsis ± shock) | Up to Jan-2023 | RCT | 10 | 755 | OR 0.51 (0.37-0.69); I² = 0% | MD -0.05 (-1.69; 1.58) → ns | MD -27.9 (-49.8; -5.9); I² = 95% | INPLASY | Cochrane RoB 2 | Egger (sec.) | Fixed (mort.); Random (sec.) |
| Martimbianco 2022 | Vit C monotherapy vs placebo (RCT) | Adults (sepsis/shock) | Up to 2021 | RCT | 4 | (subset within total) | RR 0.60 (0.45-0.80); I² = 0% | — | — | PROSPERO | Cochrane RoB 2 | Funnel | Random-effects MA |
| Cai 2022 | Vit C (RCT + cohorts; septic shock) | Adults (septic shock) | Up to Jan-2022 | RCT + Cohorts | 7 | 1,423 | In-hosp OR 0.91 (0.76-1.08); ICU OR 0.84 (0.69-1.01) → ns | OR 0.95 (0.77-1.18) → ns | — | NR | Cochrane/NOS | Funnel | Random-effects MA |
| Na 2021 | HAT + TSA | Adults (sepsis ± shock) | NR | RCT | 9 | 1,427 | RR 0.96 (0.80-1.15); I² = 0% → ns | MD 0.65 (0.30-1.00) (SOFA change) | MD -18.16 (-25.65; -10.68) | NR | Cochrane RoB 2 | Funnel | Random-effects MA + TSA |
| Fujii 2022 | NMA / Component-NMA (GC/VitC/B1) | Adults (sepsis ± shock) | Up to 2021 | RCT | 43 | 10,257 | No differences (90 d-1 year) | — | GC component: iMD -29.8 h; ICU-LOS iMD -1.3 d | PROSPERO | Cochrane/CINeMA | NMA coherence | NMA + CINeMA |
| Assouline 2021 | HAT + TSA | Adults (sepsis ± shock) | NR | RCT | 8 | 1,335 | RR 1.02 (0.86-1.20); I² = 0% → ns | WMD -0.82 (-1.15; -0.48); I² = 0% | WMD -15 (-25; -4) | NR | Cochrane RoB 2 | Funnel | Random-effects MA + TSA |
| Wu 2021 | HAT (RCT ± cohorts) + TSA | Adults (sepsis ± shock) | NR | RCT + Cohorts | 6 | 1,559 | RCT RR 1.04 (0.81-1.33); I² = 0% → ns | RCT MD -0.86 (-1.32; -0.40); I² = 0% | RCT MD -14.68 (-24.28; -5.08) | NR | Cochrane/NOS | Funnel | Random-effects MA + TSA |
| Scholz 2021 | Vit C (duration, horizon) | Adults (sepsis ± shock) | Up to 2020-21 | RCT (± obs) | 17 | 3,133 | Longest FU (pooled): RD -0.05 (-0.11; 0.01); I² = 56%, ns | — | — | NR | Cochrane RoB 2 | Funnel | Random-effects MA |
| Li 2021 | Vit C (RCT) | Adults (sepsis ± shock) | Up to 2020-21 | RCT | 10 | — | RR 0.83 (0.65-1.05); I² = 40% → ns | MD 1.32 (0.80-1.85); I² = 88% | — | NR | Jadad/Cochrane | Funnel | Random-effects MA |
| Kanchanasurakit 2021 | IV Thiamine monotherapy | Adults (septic shock) | NR | RCT + Cohorts | 3 (RCT) | 645 | OR 0.96 (0.72-1.28); I² = 0% → ns | — | — | NR | Cochrane/NOS | Funnel | Random-effects MA |
| Fong 2021 | Component-NMA (GC/VitC/B1) | Adults (sepsis ± shock) | NR | RCT | 33 | 9,898 | Ascorbate alone: RR 0.74 (0.57-0.97) (<90 d); GC+fludro: RR 0.89 (0.80-0.99) (<90 d) and RR 0.89 (0.82-0.98) (≥90 d) | — | GC comp: MD -0.96 d (shock reversal) | NR | Cochrane/CINeMA | NMA coherence | NMA + CNMA |
| Somagutta 2021 | HAT (RCT ± cohorts) | Adults (sepsis ± shock) | NR | RCT + Cohorts | NR | 67,000 (cohorts) | Hospital (RCT) RR 0.99 (0.83-1.18); ICU (RCT) RR 0.77 (0.51-1.15), I² = 58% → ns | WMD -0.42 (-1.20; 0.35); I² = 66% → ns; RCT sensitivity: WMD -0.72 (-1.30; -0.14) | WMD -25.49 (-34.37; -16.61); I² = 46% | NR | Cochrane/NOS | Funnel | Random-effects MA |
| Feng 2021 | IV Vit C monotherapy (RCT) | Adults (sepsis ± shock) | NR | RCT | 9 | 584 | OR 0.60 (0.42-0.85) (28 d; fixed) | SMD -0.33 (-0.87; 0.20) (ICU-LOS) | SMD -0.88 (-1.48; -0.29) (vasopressor dose) | NR | Cochrane RoB 2 | Funnel (visual) | Fixed/Random |
| Ge 2021 | Vit C + thiamine (RCT) | Adults (sepsis/shock) | NR | RCT | 7 | 868 | In-hosp OR 1.11 (0.79-1.56); I² = 0% → ns | WMD 0.83 (0.27-1.38); I² = 0% | WMD -17.73 (-29.76; -4.98); I² = 45% | NR | Cochrane RoB 2 | NR | Random/Fixed |
| Zayed 2022 | HAT (RCT) + TSA | Adults (sepsis ± shock) | NR | RCT | 6 | 839 | Long-term RR 1.05 (0.85-1.30); ICU RR 1.03 (0.73-1.44) → ns | MD -0.92 (-1.43; -0.41); I² = 0% (day 3) | — | NR | Cochrane RoB 2 | Funnel | Random-effects MA + TSA |
| Qian 2020 | IV Thiamine monotherapy (RCT) | Adults (septic shock) | NR | RCT | 4 | 592 | OR 0.87 (0.62-1.21); I² = 0% → ns | SMD 0.02 (-0.18; 0.21) → ns | SMD -0.02 (-0.33; 0.30) → ns | NR | Cochrane RoB 2 | NR | Random-effects MA |
| Shi & Tie 2020 | HAT (mini-MA) | Adults (sepsis/shock) | NR | RCT + Cohorts | 4 | — | RCT RR 0.92 (0.69-1.24); Cohorts RR 0.46 (0.25-0.86) | RCT WMD -1.02 (-1.31; -0.73) | RCT WMD -21.77 (-29.26; -14.29) | NR | Cochrane/NOS | NR | Mini-MA (letter) |
| Wei 2020 | Vit C (RCT + retrospective) | Adults (sepsis) | Up to 2020 | RCT + Retrospective | 4 | 1,671 | 28 d OR 0.84 (0.43-1.65); ICU OR 0.79 (0.51-1.25); In-hosp OR 0.76 (0.47-1.22) → all ns | — | SMD -0.873 (-2.269; 0.523) → ns | NR | Cochrane/NOS | Funnel | Random/Fixed-effects MA |
